# Systematic review on somatization in a transcultural context among teenagers and young adults: Focus on the nosography blur

**DOI:** 10.3389/fpsyt.2022.897002

**Published:** 2022-07-25

**Authors:** Mathilde Salmon, Jordan Sibeoni, Aurélie Harf, Marie Rose Moro, Maude Ludot-Grégoire

**Affiliations:** ^1^APHP, Hôpital Cochin, Maison de Solenn, Paris, France; ^2^Faculté de Médecine, Université Paris-Saclay, Le Kremlin-Bicêtre, France; ^3^Service Universitaire de Psychiatrie de l'Adolescent, Centre Hospitalier d'Argenteuil, Argenteuil, France; ^4^ECSTRRA Team, UMR-1153, Inserm, Université de Paris, Paris, France; ^5^Université Paris-Saclay, UVSQ, Inserm, CESP, Team DevPsy, Villejuif, France; ^6^Université de Paris, PCPP, Boulogne-Billancourt, France

**Keywords:** somatic complaint, somatization, transcultural, nosography, adolescence, discrimination, diagnostic delay

## Abstract

**Aims:**

Somatic complaints are a frequent cause for consultation in primary care. In a transcultural context, somatic complaints are typically associated with psychological distress. A recent review about somatic symptom disorders in adolescence showed some nosographic heterogeneity and outlined various etiological hypotheses (traumatic, environmental, or neurologic), separate from the cross-cultural considerations. Migrants' children encounter specific problems involving cultural mixing-issues of filiation (familial transmission) and affiliation (belonging to a group). This paper aims to provide a systematic review of somatization in transcultural contexts among teenagers and young adults, aged 13 to 24, over the past decade.

**Methods:**

This review adheres to the quality criteria set forth by the PRISMA guidelines (Preferred Reporting Items for Systematic Reviews and Meta-Analyses). Two authors queried three English databases (Medline, PsycInfo, WebOfScience) about somatization in transcultural contexts (migrant or non-Western population) among teenagers (13–18), young adults (19–24), or both. The methodological process comprised articles selection, data extraction, and then the analysis of emerging themes. Setting selection criteria to limit the transcultural field was difficult.

**Results:**

The study analyzed 68 articles. We present a descriptive analysis of the results, centered on three main themes. First, the literature highlights a nosographic muddle reflected in the combination of anxious and depressive symptoms together with the highly variable symptomatology. Second, discrimination issues were prevalent among the migrant population. Lastly, the literature review points out possibilities for improving a care pathway and reducing the diagnostic delay induced by migrants' hesitancy about Western care and the recurrent use of inappropriate diagnostic criteria.

**Conclusion:**

This review discusses the links between the nosographic muddle described here and the diagnostic delays these patients experience and raises concerns about rigid diagnostic compartmentalization. The work of the psychiatrist Frantz Fanon is here useful to understand externalized symptoms resulting from physical and psychological confinement. Discrimination issues raise questions about the cultural counter-transference health professionals experience in dealing with young migrants. Defining healthcare professionals' representations about somatic complaints in a transcultural context might be a fruitful path to explore in future research.

**Protocol PROSPERO registration number:**

CRD42021294132. Available from: https://www.crd.york.ac.uk/prospero/display_record.php?ID=CRD42021294132.

## 1. Introduction

Somatic complaints are a frequent reason for consultation in primary care, especially as the expression of suffering can be embodied in many forms and at all stages of life. The terms used to define them vary with the medical specialty, their presentation, and their severity: functional symptoms, somatization, psychosomatic disorders, medically unexplained symptoms, etc. (1). The American Psychiatric Association's Diagnostic and Statistical Manual (DSM-5) defines the group “Disorders with somatic symptoms and derivatives,” which includes: somatic symptom disorder, excessive fear of having an illness (illness anxiety disorder), functional neurological disorder (formerly conversion disorder), psychological factors influencing other medical conditions, factitious disorder, and other related disorders (2).

Studies of adults report a variety of prevalence rates of somatization worldwide. Although some studies (3, 4) find a globally constant prevalence, others highlight differences in frequency (4, 5), intensity (4) or presentation (6) in certain populations, according to the patient's geographical origin [Latin America—(4), parts of Africa—(4), India—(6), and Asia—(6)] or migration pathway [refugees—(4, 7)].

This somatic expression of distress, which appears to be more visible among so-called non-Western populations and those of migrant origin, is thought to be explained partly by external factors, specifically, their greater exposure compared with nonmigrants to stressful events, negative emotion, and psychological distress (7).

The literature on somatization in younger people is also growing. Somatoform disorders are estimated to affect between 4 and 12% of the child and adolescent population (6, 8) and are notably higher among those visiting doctors, varying between 25 and 50% (9). It is most often expressed as recurrent pain, including especially abdominal pain and headaches (6, 9)—and leads to a medical consultation in one third of cases (6). A recent French review of the literature on somatic symptomatology disorders in adolescence emphasizes the heterogeneity of designations, with DSM-5 terminology still not widely used (8). Etiological hypotheses in this population subgroup are varied: from trauma to family or social sources to neurocognitive theory (8).

Regardless of the patient's age, primary care physicians often find these consultations challenging (1, 10, 11). Ludot et al. note that young patients and their families often appear to be fruitless wandering medical pathways, at strong risk of diagnostic delay (8). If the parents are migrant patients, the practitioner may be still further confused by the uniqueness of the somatic clinical presentation, and this could have significant consequences on the patient's care. Many studies have observed the impact of racism on health, access to care, and health-related stress (12–14). These data are important to consider when analyzing our results at a more meta or higher level.

Further study of the state of the art on somatic symptomatology disorders in adolescence requires an examination of this subject of somatization in young people in a transcultural context. We now know that cultural elements must be taken into account in the diagnostic process, as DSM-5 notes with its glossary of “cultural concepts of distress,” which include cultural syndromes, cultural modalities of distress, and cultural explanations or perceived causes (15). One important question is if this cultural recognition affects care and especially it improves care or outcomes. The children of migrant families present additional complex issues entangled with the migration process. Moro et al. stress that adolescents of migrant parents are confronted with a challenge of métissage, that is, of appropriately mixing or combining their filiation (parental transmission) and affiliations (belonging to a group) (16). In their quest for self-identification, young people question the parental role and are confronted with the question of with whom they belong. The complexity and conflict involved in these questions related to transmission increases with the distance between the cultural codes of the parents' country and those of the host country.

Our aim is to conduct a systematic review of the English-language literature on somatization in transcultural contexts among young people aged 13–24 years.

## 2. Methodology

Our methodology follows the PRISMA (Preferred Reporting Items for Systematic reviews and Meta-Analyses) recommendations, and our search algorithm, combining “Mesh terms” and free text, is described in the Appendix.

Our search strategy called for us to use it to search three databases: MEDLINE, PSYCINFO, and WEBOFSCIENCE. We next checked the bibliographies of the resulting articles to include additional relevant references. In addition, we registered this review with PROSPERO, an international database of systematic reviews (registration number: CRD42021294132).

The eligibility criteria are as follows:

- Articles published in English, filtered over the last 10 years [January 2010-January 2020].
- Theme: Somatization in adolescents and young adults in a cross-cultural context. Exclusion of eating disorders (which belong to a specific DSM category) and dysmorphophobic disorders, which now belong to the Obsessive Compulsive Disorders of the DSM-5. The transcultural criterion was met by selecting articles on populations of migrant origin or “non-Western” populations in which the topic of culture was addressed.
- Covering participants: adolescents alone (filter; “adolescent 13–18 years”), or young adults alone (filter; “young adult 19–24 years”), or a combination of both. Articles in which the average age of the study sample was less than 13 or more than 25 were excluded.
- Types of studies: case reports, randomized controlled trials, non-randomized controlled trials, prospective cohort studies, retrospective studies, and qualitative studies. Commentaries, editorials, opinion pieces, and purely descriptive articles were excluded.

Data extraction: Two authors (ML and MS) independently reviewed all titles and abstracts according to the inclusion criteria and then analyzed the full texts. Two other authors (JS and AH) intervened in case of discrepancies to reach a consensus. ML and MS then extracted the following characteristics: references (year of publication, authors, country, journal, title), methodology used, objectives, and the main epidemiologic, clinical, etiologic, and therapeutic findings.

Difficulties in selection arose during the screening procedure due to the multiple terms used in the literature in reference to somatic complaints. After discussing this issue with the research group, the authors chose to retain the following terms to cover as broadly as possible the terms used in the scientific field: “somatoform disorder(s),” “somatization,” “somatic symptom(s),” “medically unexplained symptom(s),” “functional disease(s),” “functional symptom(s),” “somatic symptom disorder,” “illness anxiety disorder,” “conversion disorder,” “functional neurological symptom(s),” and “factitious disorder.” The transcultural context was also difficult to delimit. We have chosen to consider all the following terms as belonging to transcultural contexts: “cross cultural comparison,” “cultural characteristics,” “cultural diversity,” “culturally competent care,” “cross-cultural,” “transcultural,” “transients and migrants,” “migrant,” “refug*,” “migration,” “cultur*,” and “immigrant.”

## 3. Results: Descriptive synthesis of the data

**Figure 1:**
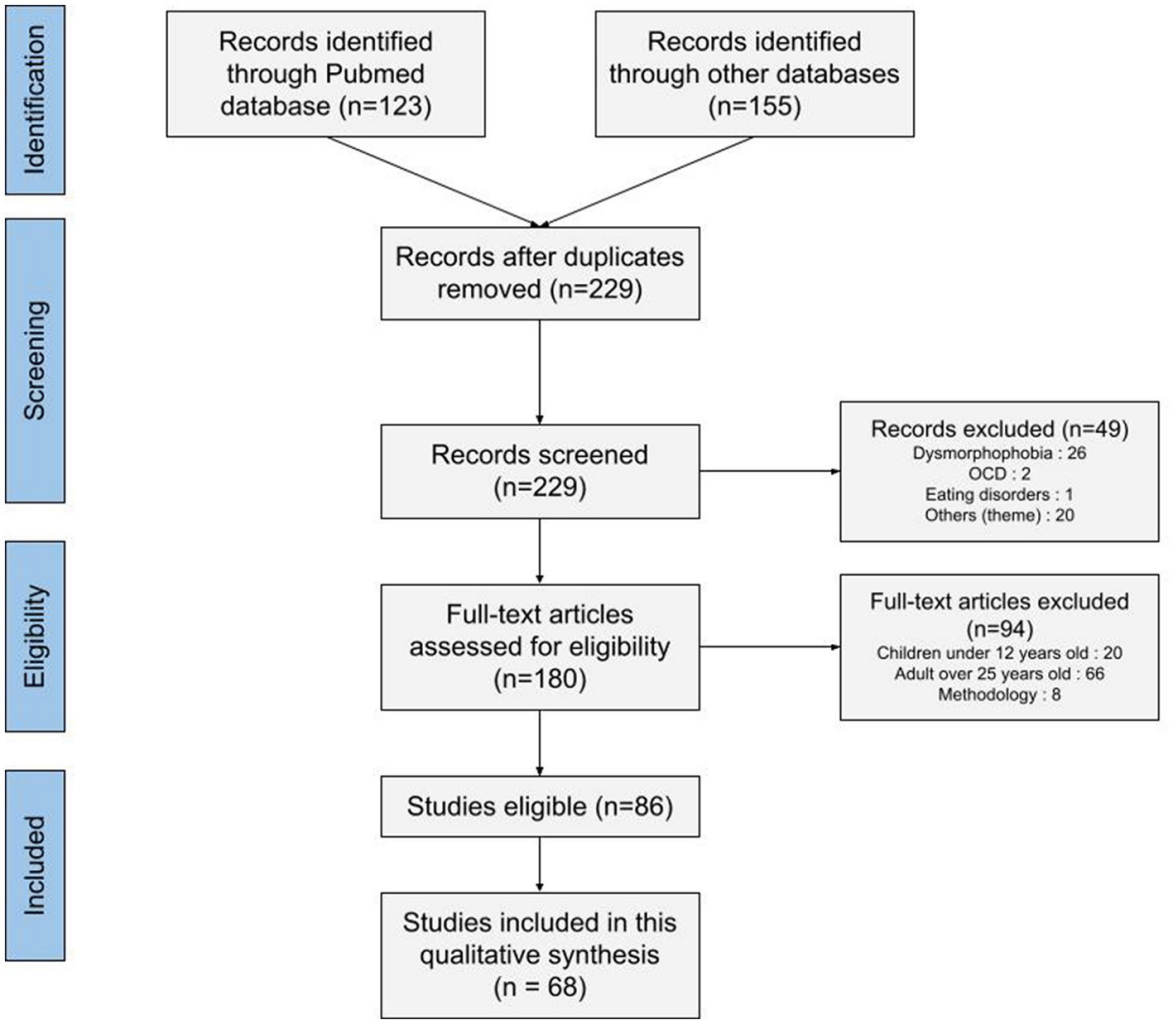
PRISMA flow diagram for the systematic review.

Among the 278 studies retrieved by the search terms, 86 relevant articles were selected. Figure 1 shows the selection process. We chose, in view of the number of articles and emerging themes, to present three central themes in this article, based on a descriptive synthesis: an ill-defined nosography, issues of discrimination, and ideas for healthcare in view of the diagnostic delay. These themes appeared in 68 articles.

### 3.1. An ill-defined nosography

In this literature review, we found a fairly homogeneous use of terms concerning the somatic expression of distress; the most frequent were “somatic symptoms,” “somatic complaints,” and “somatization.” The DSM-IV term “somatoform disorder” is found in only a few articles (17–21), while the DSM-5 term “somatic symptoms and related disorders” was not used at all. These terms are mainly related to the specific definition and term used in the assessment scales the authors chose, e.g., the Symptom Checklist 90 (SCL-90), the Brief Symptom Inventory (BSI), which is a short version of the SCL-90, the Somatic Symptoms Scale (SSS-8), and the Youth Self Report (YSR), which includes a subscale labeled “somatic complaints.” The precise meaning or implications of the terms used in reference to somatic symptoms was generally difficult to determine, because of both the frequent overlap with anxiety and depression and the highly variable clinical presentation.

#### 3.1.1. Entanglement between anxiety and depression

Two broad categories of studies were identified: those focused on somatization as an entity in its own right, as defined by the DSM diagnostic classifications, and those considering somatic complaints as part of a broader picture, such as depression (22–29), or of a still broader entity comprising the so-called “internalizing symptoms,” which encompass anxiety, depression, and somatization (24, 30–38).

Comorbid anxiety-depression is indeed strongly present in this review. The studies reported some specificities in non-Western populations, in particular, a specific chronological order in its association with somatic symptoms.

The study by Hamamura's Japanese team compared 155 Japanese students and 176 American students. It showed that depression is strongly correlated with somatic symptoms in both countries, regardless of culture or gender (39). Romero-Acosta et al. confirmed this finding in two adolescent populations in Spain, comparing Latin American immigrants with native Spanish youth. They showed that despite the migrants' higher levels of depressive symptoms, the groups did not differ for somatic symptoms (35).

Departing from the stereotype that Asian populations tend to have more somatic depression, a study of the validity of the Beck Depression Inventory (2nd edition) comparing undergraduates of Chinese and European heritage in Canadian universities found that those from Chinese families had higher scores on cognitive symptoms of depression (23). A validation study of the Korean version of the Mood and Feelings Questionnaire (MFQ) made the same observation: compared with Westerners, depressed Korean youth (mean age: 14.2 years) were more likely to complain of cognitive and emotional rather than somatic symptoms (40).

A longitudinal study of the development and interrelations between internalized symptoms in Canadian youth from age 11 to 17 years showed complex associations. Anxiety and depression consistently predicted somatization. Anxiety also had an indirect effect on somatization *via* depression. According to the accounts of these young people's parents, there are several bidirectional effects between anxiety and depression and between depression and somatization. Parents who were White and or high-income parents reported lower levels of internalized symptoms in their children (31). The same longitudinal relations were explored in an article that focused on Vietnamese, Vietnamese-American, and European-American adolescents. It also highlighted a cultural difference in the chronological sequence of onset of internalized symptoms: in Vietnamese and Vietnamese-American youth, somatic complaints predicted anxiety. The authors point out that the similarities between the Vietnamese and Vietnamese-American groups offer evidence of the importance of the ethnocultural heritage of symptoms (24).

#### 3.1.2. Variable semiology

Most of the complaints described in this review involved pain, particularly headaches (41–46), abdominal pain (41, 43, 45, 46) and asthenia (20, 43, 47).

Like Western origin, female gender is a risk factor for somatization (31, 35, 36, 43, 46, 48–55). There is also evidence that the risk of somatization increases with age (44, 56).

The literature shows specificities in the so-called “non-Western” population. A Japanese study, for example, reports more frequent physical symptoms and psychiatric disorders in Japanese children and adolescents than in Swedish youth (57). In the Middle East, Iranian youth tend to have more severe psychogenic nonepileptic seizures (PNES) than youth of 4 other nationalities (Brazil, USA, Canada, and Venezuela) (58). An Austrian study also found variable trauma-related symptom patterns depending on origin: Turkish-speaking adolescents and young adults have more behavioral problems associated with their trauma, while Austrians described sleep disturbances and weight loss more often (59). Similarly, specific conditions may be more frequent in some countries than others: in Japan, up to 71% of children and adolescents with a psychosomatic disorder are reported to have orthostatic intolerance, which is thus one of the most common psychosomatic disorders in that country (57).

Psychosomatic disorders also affect populations of migrant origin. In Switzerland, for example, Michel and his team reported that a migratory background is a risk factor for additional somatization in young cancer survivors (OR = 4.1) (60). Symptoms may also vary in intensity: a higher number of physical symptoms has been observed in refugee populations (61–64) and ethnic minorities (65). On the other hand, a protective factor has been identified in the population of Asian migrants: ethnic identity, which includes the maintenance of cultural values and practices and a sense of belonging (66).

These initial findings show that the distress expressed by the body in young people presents itself in a wide variety of ways, which may change across cultures. In particular, the chronological presentation of somatization is associated with anxiety and depression, but in sequences that differ depending on origin.

The idea of somatization is preponderant among migrants, essentially among refugees and minorities, but the evidence is not unequivocal. Two studies of Western youths produced somewhat contradictory results: they appear more likely to express somatic complaints in the context of anxiety-depressive disorders (23, 24) while their parents appear to report them less often (31).

### 3.2. Discrimination issues

There was one major commonality between the different migrant populations referred to in this review: they all face the issue of acculturation. According to Berry, acculturative stress refers to the trade-offs that migrants face as a result of differences between their home and host cultures [cited by (36)]. This stress results from several factors, including the need to learn new and sometimes confusing cultural rules and expectations, experiencing prejudice and discrimination, and managing the conflict between maintaining elements of the old culture while incorporating those of the new, according to Berry and Suàrez-Orozco (36). Rumbaut and Mahalingam observed that acculturative stress also stems from negative stereotypes and attitudes that the host culture may hold toward migrants [cited by (36)].

Discrimination was the phenomenon most often discussed in the articles we reviewed. A 4-year longitudinal study found an association between perceived discrimination and the expression of somatic symptoms in young students of immigrant origin in the USA (67). Another study, conducted in Israel with migrant adolescents (from the former Soviet Union and Ethiopia) investigated the relations between the experience of discrimination and externalized behaviors (aggression and substance abuse). It found that discrimination reduced the young migrants' sense of identity as part of the host country and led to increases in “psychosomatic symptoms,” aggression, and substance abuse. The authors found that psychosomatic symptoms mediate the link between discrimination and externalized behaviors and that this mediating role differs by origin: it was partial for the adolescents from the former USSR and total for the Ethiopian youth (68).

Another study examined how ethnic composition in US schools, as measured by the percentage of non-Hispanic White students in a school, affects depressive and somatic symptoms in a representative sample of 18,419 US adolescents, and whether the association differs by ethnicity. After controlling for individual and school characteristics, the analysis indicated that the likelihood of black students experiencing somatic symptoms increased with the percentage of white students in their school. This interaction between somatic symptoms, Black ethnicity and the school's ethnic composition was no longer significant, however, after controlling for the effects of feeling discriminated against and school attachment (69).

In New York, Ong and his team found that 78% of Asian Americans experienced daily racial microaggressions and that somatic symptoms increased with their number (70). The same was true among Chinese-American families in California, where adolescents' experiences of discrimination were linked to an increase in somatization. It is important to note that in these families, higher conflict exacerbated the negative effects of discrimination, while greater family cohesion mitigated its negative effects (71). The same authors reported similar findings with a larger sample in 2012 and pointed out that the acculturation process gives rise to conflicts between parents and adolescents over everyday issues and deeper cultural values (72). In contrast to everyday conflicts, however, the levels of acculturation-based conflicts are not similar across ethnic groups. Lee and Liu have found that Asian Americans report higher levels of acculturation-based conflict than Latinos or European Americans [cited by (72)].

Discrimination and associated acculturative stress are prominent in this review of somatization. Everyday challenges for young people of migrant origin raise the issue of ethnic identity in families. They are a source of intrafamily conflicts between young people and their parents that are often expressed through somatic complaints.

### 3.3. Care must be improved

Many improvements remain to be made in patient care for young people with somatic symptoms. The initiation of their treatment is sometimes delayed, and the provision of care is more limited for some patients. An Austrian study looked at the medical records of young people with a history of trauma of various kinds (“trauma-related disorder”) and compared the data between patients of Turkish origin in Austria and native Austrians. The natives received psychiatric and psychotherapeutic treatment more often (32.7%), whereas Turkish-speaking patients mainly received only psychiatric treatment (59). The delay in diagnosing psychological distress has often been pointed out.

#### 3.3.1. Delayed diagnosis

The longer delays in diagnosis for non-Western patients expressing their distress through the body are well known and widespread. For example, two studies of patients with PNES (psychogenic nonepileptic seizures) noted diagnostic delays of 4–5 years. One article looked at patients from 5 different countries and reported a delay of about 4 years from the first symptoms (at a mean age of 12.1 years ± 3.2) to diagnosis (mean age: 16.7 years ± 7.5) (58). The same team studied data from 314 patients in Iran aged 17 or older at PNES onset and reported a delay of 5 years between the first symptoms and diagnosis (mean age at onset: 23 years, and mean age at diagnosis: 28 years) (73). However, in the group of pediatric patients, that is, aged 16 years and younger, the diagnostic delay, in contrast, was approximately 1 year (74).

A case report of a 24-year-old Pakistani patient living in Australia with a Dharan picture highlights a prolonged medical history. This is a cultural syndrome according to the DSM-5, described as a particular set of symptoms with a common understanding of causality shared by specific cultural groups or communities. Here the patient presented with multiple symptoms with epigastric pain, back pain, nausea, anorexia, dehydration, bloating, and fluctuating constipation. Only after four consultations in the emergency department and several examinations (abdominal ultrasound, chest X-ray, abdominal and pelvic CT scans) and treatment options was she seen by a psychiatrist accompanied by an interpreter (75).

Evidence explaining these delays comes from Aggarwal et al. who conducted a systematic review of practitioner communication patterns and ethnic minority patient engagement in mental health care. They concluded that the primary cause of delay in initiating treatment was the patient's view that the care system could not help them. The second reason was the patient's own somatic interpretation of a mental health problem. This attitude was more common in patients with limited English language skills (in Australia) and in immigrants from South Asia, East Asia and the Dominican Republic living in the USA. The third reason was that stigma interfered with care. Barriers to treatment adherence once it began included discomfort discussing emotions with a stranger, preference for a different communication style from the practitioner, and concern about the clinician's power (76).

Another hypothesis to explain the delay in diagnosis is the use of Western diagnostic criteria, which are less adapted to certain populations.

An American study of adolescents on the chronological order of internalized symptoms shows that precursor symptoms of depression differ according to patients' origin. More specifically, although most often in the USA, anxiety symptoms predict and thus can help prevent depression, young people of Vietnamese origin (whether born in Vietnam or in the USA) initially present more somatic complaints rather than anxiety symptoms (24).

Two articles question the assessment of depression by standardized tools among non-Western populations and highlight the need to modify some diagnostic criteria for greater inclusivity.

One paper investigated the utility of the DSM-V and ICD-10 diagnostic criteria for major depressive disorder (MDD), originally developed in the West, for Indian clinicians treating Indian adolescents with depressive symptoms. Clinicians found the majority of DSM-5 and ICD-10 criteria for MDD to be useful, but further identified additional markers of depression in Indian youth (interpersonal conflicts and problems, disturbed school functioning, anger-related symptoms, anxiety-related symptoms, and additional somatic complaints not included in DSM-5 or ICD-10) (25).

Another study tested the psychometric properties and factor structure of the Center for Epidemiological Studies on Depression (CES-D) scale in Jordanian adolescents aged 13–17 years. The results revealed that the construct of depression among Arab adolescents, as measured by the CES-D, differs from that of other ethnic groups. A two-factor rather than four-factor scale provides a reasonably better fit: factor 1 combined items on depressive affect, somatic complaints, and interpersonal problems, and factor 2 included the remaining four positive affect items. The authors stressed that professionals must pay attention to the different expressions of depressive symptoms between ethnic groups to avoid errors (26).

#### 3.3.2. Management approaches

Fourteen articles looked at the care of somatization in a cross-cultural context. Our results highlight the use of a multidimensional approach: medical, either Western or traditional (75), psychological, pharmacological (59, 77) but also socio-educational (50, 57).

For example, Japanese pediatricians recommend multidisciplinary management by the health and education systems for orthostatic intolerance in young people. They recommend parental guidance, psychotherapy, psychosocial intervention, pharmacological and nonpharmacological symptomatic treatment (from physical maneuvers to restoring sleep/wake rhythms) (57).

This review identified mindfulness as a specific technique discussed in several articles (78–80). It is defined as a psychological process of emotion regulation (78) and reflects the experience of paying attention without mental judgment to the unfolding of the present moment (79).

A study of 116 Asian American participants in the United States (with an average age of 19.89 years) who responded to an online survey, found that mindfulness and psychological rigidity were uniquely and separately related to somatization, depression, and anxiety after controlling for age and gender. Greater psychological rigidity was associated with greater somatic, depressive and anxiety symptoms, and mindfulness with fewer or less serious somatic, depressive, and anxiety problems (78).Mindfulness was also studied in an article about stress resilience in Iranian students. Results showed that it was a negative predictor of physical symptoms (assessed by the 20-item Symptom Checklist) (80).

Similarly, another article describes the care of functional somatic symptoms in a Danish pain clinic. The aim is to shift the focus from physical pain to self-awareness and body awareness and to address dietary rules for a healthy lifestyle (81). A similar strategy was proposed for the management of a 17-year-old Indian patient with another cultural syndrome, Dhat syndrome, with the aim of shifting the focus from the symptoms to healthier lifestyle habits (82).

Another type of treatment mentioned is trauma-focused cognitive-behavioral therapy. It is used in the care of young refugees in Germany and has resulted in significant improvement in physical symptoms (assessed by the PHQ 15) (83). Concrete improvement of the environment is particularly pointed out as a therapeutic lever in the refugee populations living in camps. In a camp for adolescent refugees in Turkey, changes in living conditions, learning opportunities, and medical care were shown to lead to a decrease in somatization (50).

Traditional therapeutics, such as Ayurvedic medicine, herbal medicine, etc. especially in culture-related syndromes, are a treatment option to consider with some patients (75).

A key point in the care of migrant patients seems to be the provision of a culturally relevant approach. In their review of care for ethnic minorities, Aggarwal et al. conclude that improvements could be made by incorporating patients' views of the disease into treatment and targeting stigma. Clinicians can improve treatment engagement by using plain language, tailoring communication to patient preferences, discussing differences, and demonstrating positive affect (76).

ONonetheless, a study of young people currently or recently living away from home in the USA raises important questions about these suggestions. A survey of the feelings of professionals providing therapy for these families showed that many of them found it more difficult to work with minority families. The therapists perceived the therapeutic alliance was better when the parents belonged to a white/European-American ethnic group and when they obtained low BSI (the Brief Symptom Inventory, a short version of the SCL-90) scores, including the somatization subscale (84).

The combination of communication difficulties, patient and care provider representations, and inappropriate diagnostic tools creates substantial barriers to treatment. Delays in diagnosis and treatment increase for these young people, and adherence is compromised. This review highlights multiple avenues of treatment that, while tending toward a multidimensional approach, remain unspecific.

## 4. Discussion

This review of the English-language literature made it possible to extract 68 articles dealing with somatization in a transcultural context. The descriptive synthesis of these articles highlights the difficulties inherent in a vague and ill-defined nosography, the frequent association of problems of discrimination, and finally, significant diagnostic delays and aspecific healthcare approaches. We will first discuss the links between discrimination, ill-defined nosography, and healthcare delays. Secondly, we will see how the work of Frantz Fanon and its studies can shed light on the issues of discrimination associated with somatic symptoms.

### 4.1. Discrimination, ill-defined nosography and delays in care

It is difficult to name the bodily expression of psychological distress in medical diagnostic terms. This is not only true in the transcultural context. A systematic review of the literature on the subject revealed a great heterogeneity of names and very little use of the DSM-5 terminology: “disorder with somatic symptoms” (8). Similarly, the borderlines of this diagnosis, in psychopathological terms, are unclear. In particular, it is very strongly entangled with anxiety and depressive symptoms (85–89). The actual content of a diagnostic term to embody medically the somatic expressions of distress remains unclear.

Contact with the transcultural field makes these terms appear even more impoverished. In particular, no study in this review mentions the DSM-5 diagnosis, while only 10 referred to it in a review of this topic outside the transcultural field (8). In non-Western and migrant populations, it is only a question of “somatic complaints/symptoms” and “somatization.” Could the additional difficulty in naming come from a loss of our diagnostic reference points in a transcultural context? Might issues of discrimination, highly present in this review, have something to do with this additional difficulty in naming?

This review points to significant delays in diagnosing psychological distress, and vague and impractical approaches to treatment. The therapist's countertransference must then be questioned, particularly in relation to the patient's culture. This specific countertransference is defined as “the set of reactions of a person to the encounter with another person from another culture who enters into a relationship with him/her” (90). It is essential to be aware of “cultural coding,” i.e., the divergence of clinical manifestations and symptoms from one culture to another, and of the necessary “decentering,” that is, “to not interpret the unknown in terms of the known” (91). This countertransference is especially important to analyze, because there are necessarily traumatic elements (92).

A qualitative study of the experiences of health professionals would be useful to understand how they perceive the expression of somatic symptoms in the clinical populations they treat, specifically adolescents and young adults, according to the patient's cultural history. In a more cognitivist reading, it might be considered a question of analyzing these professionals' “implicit biases,” particularly in a transcultural context. That is, a doctor might systematically—illogically and irrationally—determine the importance of symptoms of the same nature according to characteristics, often cultural, of the person presenting them. Accordingly, a systematic review of 15 studies investigated the implicit racial and ethnic biases among health professionals (93) thought to be responsible for the inequalities in access to care, quality of care received, and health outcomes that people of color face in the USA. Although some associations between implicit attitudes and medical outcomes were not significant, the results showed that some of these attitudes were significantly related to patient-prescriber interactions, treatment decisions, treatment adherence, and medical prognosis. Are we dealing with racial prejudice (which can be explored through cultural countertransference or implicit bias) or with racialization/racization of patients? Racialization or racization in health care can take the form of “prejudice, clinical uncertainty, beliefs, stereotypes of care” on the part of health professionals (94). In addition, “communication problems between patient and provider” can contribute to health disparities (94).

Furthermore, we know that it is important to use the patient's mother tongue to access emotional subtleties as well as representations and body expressions. Thus, the use of interpreters is essential for diagnosis (95). But we also need to adapt our modalities of symptomatic assessment to the patient's culture—and do so without ignoring the transcultural principle of psychic universality. The Temas (Tell-Me-A-Story) developed in 1988 in New York by the Italian psychologist Giuseppe Costantino et al. (96) is an illustration of this: this projective and narrative test was invented in response to the observation that conventional tests did not always allow minorities living in the United States to verbalize their intrapsychic conflicts, as it was sometimes difficult for them to identify with the characters represented. Similarly, Fanon, a psychiatrist from Martinique who directed the psychiatric department of the Blida hospital in Algeria, sought to reconstruct personality tests or thematic perception tests that represented scenes that were incomprehensible to the local populations because of the inadequacy of the cultural references of the images presented (97). When questioning the symptoms coming from the body, it is necessary to know the representations of the body in the patient's culture.

### 4.2. Illumination from the work of Frantz Fanon and subaltern studies

The mediating role of somatic expressions between immigrant discrimination and externalized symptoms appears to be a particularly interesting theme observed in this review. One important theme of Fanon's work was precisely the link between alienation and violence. He contributed to the understanding of the psychology of oppression (98), in both his psychiatric and political reflections on the relations between White and Black individuals in the colonialist context of the time. He considered assimilation (which he had experienced in his natal Martinique and had fled) a form of alienation (99). According to Fanon, colonization freezes, blocks, and compartmentalizes the colonized; and for him any compartmentalization, in either the body or in thought, produces alienation. He thus analogized the impact of confinement in an asylum on psychiatric alienation and the impact of colonialism on historical alienation. This parallel is carried forward in an article dealing with migration in Italy (100), highlighting the impact of nationalist and xenophobic language on migrants and in particular on the violent radicalization of second- and third-generation immigrants. Compartmentalization in thought would thus produce physical and psychological effects of uneasiness.

Fanon presents himself as a man who has experienced how some people in a colonized society look at a man they call a “man of color” (101). Elaborating a phenomenology of colonized consciousness, describing the path of this consciousness that passes “from the desire for assimilation to negritude” [chapter 5 “The Fact of Blackness” (99)].

He speaks of a “corporeal schema, assailed at various points” and which “crumbled, its place taken by a racial epidermal schema.” He describes a day, when he had “to meet the white man's eyes…completely dislocated, unable to be abroad with the other, the white man, who unmercifully imprisoned me, I took myself far off from my own presence, far indeed, and made myself an object.” And further on he adds “My body was given back to me sprawled out, distorted.” (102). Violence is thus thought of as a reaction to a situation of confinement. We then witness an embodiment of the colonial gaze, internalized by the colonized, in the form of “muscular and mental rigidity” and at a certain point, a “motor crisis of dissolution” appears: this is when violence occurs (103). Through the incessant parallels that Fanon, the militant, draws to colonialist alienation, Fanon the psychiatrist illuminates his clinical observations: similar psychic and bodily effects on the human subjected to compartmentalization. And discrimination can indeed be considered to compartmentalize the person subjected to it.

The Subaltern Studies Group was a group of South Asian scholars interested in the postcolonial and post-imperial societies, under the leadership of Guha (104). Its work also sheds light on this topic of somatic expression of distress. The Indian philosopher Gayatri Chakravorty Spivak defined subalterns as “those who in official history are never allowed to speak” (105). The subalternist project was inflected in the late 1980s by several cross-influences; the first was Foucault's work (106), together with the epistemological relativism it engages, and the broad theme of the “power-knowledge” complex it proposes. Another influence was the work of Said et al. (107), whose influence spread rapidly in Indian historiography, taking, in particular, Subaltern Studies as its anchor. He inaugurated what was to become the postcolonial movement and demonstrated how Orientalism is a Western style of domination, restructuring, and authority. Thus, for Said, Orientalism is a scholarly but clichéd study of the East; it is also another way of dividing the world into two parts, one of which is dominant, as well as a modern way of manipulating the East for colonialist purposes.

The question of the unheard word, which makes subalterns people without identity and excludes them from discourses and representations, seems to us an important notion to discuss here, to help us understand the place that the body can take and the need to hear what it expresses. In her work “Can subalterns speak?,” a question that Spivak answers in the negative, she ends the essay with a narrative example: a situation where an Indian women (Bhuvaneswari Bhaduri) 16 or 17 years old, has tried with all her might to speak, to the point of making her suicide a message whose reach will be prevented (including by her own family which presented it as the consequence of an illegitimate love) and which will only be transmitted thanks to the philosopher's analysis of the fact that she had killed herself while menstruating. Indeed, almost 10 years after her suicide, it was discovered that “she was a member of one of the many groups involved in the armed struggle for Indian independence. She had finally been assigned a political assassination. Unable to confront the task [of assassination that had been entrusted to her] and yet aware of the practical need for trust, she killed herself” (105).

Spivak hypothesizes a rewriting of the social text of sati-suicide in an interventionist way: “she generalized the sanctioned motive for female suicide by taking immense trouble to displace (not merely deny), in the physiological inscription of her body its imprisonment within legitimate passion by a single male... The displacing gesture-waiting for menstruation-is at first a reversal of the interdict against a menstruating widow's right to immolate herself; the unclean widow must wait, publicly, until the cleansing bath of the fourth day, when she is no longer menstruating, in order to claim her dubious privilege” (105). Spivak tells her critics that some mechanisms of discrimination do not refer to subalternity, especially if they can be resolved through speech, within a hegemonic discourse. As she makes clear, “when we say ‘cannot speak', it means that, if ‘speaking' implies speaking and being heard, this possibility of a response, responsibility, does not exist in the sphere of the subaltern” (105). Thus, in the subject that interests us and inspired by this work on subalternity, we hypothesize a somatic translation occurs because verbal expressions of the experience of discrimination is impossible and cannot be heard.

## 5. Conclusion

This systematic literature review conducted in various transcultural contexts underlines the links between discrimination issues, ill-defined nosography, and delays in treatment, while at the same time highlighting the risks of rigid diagnostic compartmentalization. The issues of discrimination associated with somatic symptoms are particularly present in these articles and lead us to consider that questions need to be asked about the cultural countertransference of health professionals in contact with young migrants. A broader question than that of individual reactions, however, might be the issue of racialization in health matters. Frantz Fanon's valuable insights allow us to understand externalized symptoms as the result of a physical and psychic alienation. Subaltern studies, and particularly the writings of Spivak, allow us to hypothesize that somatic symptoms are expressions of certain discriminatory experiences that are impossible to talk about in migrant populations because they are impossible for others to hear. Additional results from this systematic review will be presented in another transcultural article. Questioning health professionals about their experiences with somatic complaints of young migrants could give us additional insight, particularly in terms of countertransference.

## Data availability statement

The original contributions presented in the study are included in the article/supplementary material, further inquiries can be directed to the corresponding author/s.

## Author contributions

MS and ML contributed to the conception and design of the study, organized the database, conducted the analysis, and wrote the manuscript. JS and AH contributed to the design of the study and supervised the analysis. MM revised the manuscript. All authors contributed to the revision of the manuscript and have read and approved the submitted version.

## Conflict of interest

The authors declare that the research was conducted in the absence of any commercial or financial relationships that could be construed as a potential conflict of interest.

## Publisher's note

All claims expressed in this article are solely those of the authors and do not necessarily represent those of their affiliated organizations, or those of the publisher, the editors and the reviewers. Any product that may be evaluated in this article, or claim that may be made by its manufacturer, is not guaranteed or endorsed by the publisher.
