# Supplementary material for: Systematic review on somatization in a transcultural context among teenagers and young adults: Focus on the nosography blur
Source: Front Psychiatry. 2022 Jul 25;13:897002. doi: 10.3389/fpsyt.2022.897002 (PMC9358691; doi:10.3389/fpsyt.2022.897002)
Supplement: Supplementary file 2 [file Table_2.DOCX]

**Appendix 2 : Boolean string in PubMed**

("somatoform disorders"[MeSH Terms] OR "somatoform disorder*" OR somatization OR "somatic symptom*" [Tiab] OR "medically unexplained symptom*"[Tiab] OR "functional disease"[Tiab] OR "functional symptom*"[Tiab] OR "somatic symptom disorder"[Tiab] OR "illness anxiety disorder"[Tiab] OR "conversion disorder"[Tiab] OR "functional neurological symptom*"[Tiab] OR "factitious disorder*"[Tiab]) AND ("cross cultural comparison"[MeSH Terms] OR "cultural characteristics"[MeSH Terms] OR "cultural diversity"[MeSH Terms] OR "culturally competent care"[MeSH Terms] OR "cross-cultural"[tiab] OR "cross cultural"[tiab] OR transcultural[tiab] OR "transients and migrants"[MeSH Terms] OR migrant*[tiab] OR refug*[Tiab] OR migration[tiab] OR cultur* [Tiab]) AND ((y_10[Filter]) AND (adolescent[Filter]))
