# Supplementary material for: Systematic review on somatization in a transcultural context among teenagers and young adults: Focus on the nosography blur
Source: Front Psychiatry. 2022 Jul 25;13:897002. doi: 10.3389/fpsyt.2022.897002 (PMC9358691; doi:10.3389/fpsyt.2022.897002)
Supplement: Supplementary file 3 [file Table_3.DOCX]

**Results table**

|  | **Source** | **Title** | **Authors** | **Journal** | **Year** | **Country (authors)** | **Study type** | **Aim** | **Age** | **Country of origin** | **Type of population** | **Results** | **Scale** | **Journal discipline** |
| --- | --- | --- | --- | --- | --- | --- | --- | --- | --- | --- | --- | --- | --- | --- |
| 1 | PubMed | [**Clinician descriptions of communication strategies to improve treatment engagement by racial/ethnic minorities in mental health services: A systematic review.**](https://pubmed.ncbi.nlm.nih.gov/26365436/) | Aggarwal NK, Pieh MC, Dixon L, Guarnaccia P, Alegría M, Lewis-Fernández R. | Patient Educ Couns. | 2016 | USA | Literature review | To describe studies on communication with clinicians and engagement/adherence of ethnic minority patients in mental health treatment | - | Minorities in these countries:  USA  Australia  England  Spain  Other European countries | 23 studies | Somatic interpretation of mental health problems = 2nd cause of delay in initiating treatment.  Avenues for improvement: integrate patients' views of the disease and target stigma. For participation: use simple language, adapt communication to patient preferences, discuss differences, and demonstrate positive affect. | - | Nursing |
| 2 | WOS | **Clinicians' Perspectives on Diagnostic Markers for Depression Among Adolescents in India: An Embedded Mixed-Methods Study** | Aggarwal, P; Raval, VV; Chari, U; Raman, V; Sreenivas, KK; Krishnamurthy, S; Visweswariah,AM | Culture, medicine and psychiatry. | 2020 | USA  India | Cross-sectional study | To investigate the validity of Western diagnostic criteria for major depressive disorder in Indian adolescents | Ado. | India | Participants = clinicians and students with at least 1 year experience in the diagnosis or treatment of adolescent depression in India | Indian clinicians identified markers of depression consistent with current diagnostic manuals.  Clinicians found the majority of DSM-5 and ICD-10 criteria for MDD useful.  Additional markers and additional somatic complaints identified: e.g. pain (headache, stomach pain). | Interview | Anthropology  Psychiatry  Social Sciences |
| 3 | PubMed | **Mental health of Syrian refugee adolescents: how far have we come?** | Akgül S, Hüsnü Ş, Derman O, Özmert E, Bideci A, Hasanoğlu E. | Turk J Pediatr | 2019 | Cyprus, Turkey | Comparative study | To reassess the mental health status (BSI questionnaire) of adolescents living in the same temporary accommodation center (TAC) in Turkey following a first study in 2016 and many measures taken to improve this problem (living conditions, learning, medical care). | 12–18 years (14,2 ± 0.83) | Syrian refugees in Turkey | Refugees | -All BSI sub-scores decreased from 2016 to 2019: somatization: 2016=1.65%, 2019=0.56%  - Girls reported significantly higher scores than boys for anxiety, depression, somatization, and overall severity index. | BSI  SSS-8 | Pediatrics |
| 4 | WOS | **Stress overload and the new nightmare for Dreamers** | Amirkhan, JH; Velasco, SE | J Am Coll Health | 2019 | USA | Cross-sectional study with anonymous self-questionnaire | To assess stress levels, symptoms, and grade point averages in Dreamers (undocumented young people who arrived in the US illegally before the age of 16) compared to other students | M = 22,2-  23,06 y | Migrant "Dreamers" | 424 students in public university in Southern California | Dreamers showed more somatic symptoms, a result mediated by stress overload. | PHQ-15 | Health services |
| 5 | PubMed | **Sexual abuse and psychogenic nonepileptic seizures** | Asadi-Pooya AA, Bahrami Z. | Neurol Sci. | 2019 | Iran | Retrospective study | To study the frequency of reported sexual abuse in patients with psychogenic nonepileptic seizures (PNES) in a Middle Eastern culture (Iran) and to attempt to characterize the association between a history of sexual abuse and the clinical features of PNES in these patients. | Age at onset = 20–23 y  Age at dx = 25-28 y | Iran | 314 patients with PNES followed at the Shiraz Comprehensive Epilepsy Center from 2008 to 2018 | 8.3% had a history of sexual abuse (lower than in the West: 32-88%). | - | Neurology |
| 6 | PubMed | **Pediatric-onset psychogenic nonepileptic seizures: A retrospective international multicenter study** | Asadi-Pooya AA, Myers L, Valente K, Sawchuk T…. | Seizure | 2019 | Iran  USA  Brazil  Canada  Cyprus  Venezuela  Argentina  England | Retrospective multicenter study | To compare various clinical characteristics of PNES with pediatric onset in patients from 5 countries. | M age dx PNES = 16.7 y (+/- 7.5)  M age et onset 12.1y | Iran, Brazil, USA, Canada, Venezuela | 229 patients admitted to epilepsy monitoring units in 5 countries | M age at dx = 16.7 y  M age at onset = 12.1 y  Similar risk factors between countries.  Different crisis semiology in different countries. |  | Neurology |
| 7 | PubMed | **Outcome of patients with psychogenic nonepileptic seizures with limited resources: A longitudinal study** | Asadi-Pooya AA, Ziyaee F. | Seizure | 2018 | Iran | Retrospective study | To study the long-term outcome of patients with PNES and the factors potentially associated with their outcome in Iran. | age at dx =24+/- 10 years | Iran | 86 patients (52 F, 32 M) | 86% did not receive appropriate psychotherapy.  Lower education, concurrent psychiatric problems, and older age at seizure onset may be barriers to improvement in seizures (criterion: absence of seizures in the last 12 months). | - | Neurology |
| 8 | Psycinfo | **Psychogenic nonepileptic seizures in children and adolescents: An international cross-cultural study.** | Asadi-Pooya, Ali A.; AlBaradie, Raidah; Sawchuk, Tyson; Bahrami, Zahra; Al_Amer, Abeer; Buchhalter, Jeffrey | Epilepsy & Behavior | 2019 | Iran  USA  Saudi Arabia  Canada | Retrospective comparative intercultural study | To compare various clinical features of PNES between young patients from Iran, Saudi Arabia, and Canada, | 16 y or less  M age dx = 13.4 y | Iran  Saudi Arabia  Canada | 51 patients (22 from Iran, 14 from Saudi Arabia, and 15 from Canada) admitted to epilepsy monitoring units. | Young patients with PNES across borders and cultures shared more similarities than differences in their demographic and clinical characteristics. | - | Behavioral Sciences  Neurology |
| 9 | PubMed | **Comparing Trauma Exposure, Mental Health Needs, and Service Utilization Across Clinical Samples of Refugee, Immigrant, and U.S.-Origin Children** | Betancourt TS, Newnham EA, Birman D, Lee R, Ellis BH, Layne CM. | J Trauma Stress. | 2017 | USA  Australia  Russia | Retrospective comparative study on data from Core Data Set | Comparing trauma exposure, psychological distress, and mental health service utilization among refugee, immigrant, and American children and adolescents | 0–21 y  M=13.07 (R)  M=13.26 (I)  M=12.11 (US) | Refugees  39.6% South or Central America  26.3% Africa  20.8% Eastern Europe  7.5% Asia  5.7% Middle East  Americans, migrants | 343 young people (60 refugees) from 56 US centers referred to the National Child Traumatic Stress Network (NCTSN) for assessment and treatment. | Trauma: Refugees > others  More important comorbidities: somatization (26.79%), dissociative symptoms, trauma, grief, phobic disorder. | Clinicianassess-ment grid | Military Medicine  Psychology |
| 10 | WOS | **Asian Indians in America: The influence of values and culture on mental health** | Chandra, RM; Arora, L; Mehta, UM; Asnaani, A; Radhakrishnan, R | Asian J Psychiatr | 2016 | USA  India | Selective review | To examine the impact of Indian culture on mental health, discuss the impact of acculturation and ethnic identity development on the mental health of Indian Americans | -  One group of teenagers | Population of Indian origin living in the USA | 120 articles on  Indian-American population | Indian culture and manifestation of depressive and anxiety disorders = higher reporting of somatization and physical symptoms in South Asians compared to Western population (Cheng, 1989; Hoge et al., 2006).  Issues of ethnic identity development (Phinney, 1990) or bicultural identity development = determining the extent to which they are 'Indian' and the extent to which they are 'American'.  Issues found in teens: parental anger over child's autonomy, depression over child not meeting parental expectations, and 2nd generation Indian-Americans seeking help due to increasing anxiety or depression over not being able to meet expectations. | - | Psychiatry |
| 11 | WOS | **Listen to Your Heart: The Cultural Shaping of Interoceptive Awareness and Accuracy** | Chentsova-Dutton, YE; Dzokoto, V | Emotion | 2014 | USA | Cross-sectional comparative study (randomized control) | Does the West African cultural emphasis on interoceptive awareness affect individuals' abilities to accurately perceive physiological changes in response to emotional stimuli? A comparison of West African and European-American youth. | 18–28 y  M. age = 22.96 (WA)  M. age=19.57 (EA) | West Africa  USA | 56 West Africans and 58 European-Americans studying at the University of Accra in Ghana and in Washington | West African youth have higher levels of interoceptive awareness but with less accuracy. | SCL 90 | Psychology |
| 12 | PubMed | **The Prevalence and Socio-Demographic Correlates of Depressive Symptoms in Early Adolescents in China: Differences in Only Child and Non-Only Child Groups** | Chi X, Huang L, Wang J, Zhang P. | Int J Environ Res Public Health | 2020 | China | Cross-sectional descriptive study | To explore the prevalence and socio-demographic correlates of depressive symptoms in young adolescents in China, as well as differences between a group of only and not-only children. | 11–15 y  M = 12,44 | China | 2,059 Chinese students in 7th grade | 34.7% had depressive symptoms  Adolescents with siblings had more severe somatic symptom scores (3.29) than only children (2.80, *P*<0.001) | CES-D | Environmental Health  Public Health |
| 13 | PubMed | **Hmong mental health needs assessment: a community-based partnership in a small mid-Western community** | Collier AF, Munger M, Moua YK. | Am J Community Psychol. | 2012 | USA | Cross-sectional study based on focus groups | To verify the mental health needs of Hmong living in a Midwestern community | Youth category  18–26 y  M=21.1 | Hmong community living in the USA | 36 people of Hmong origin | Significant mental health needs.  Participants described problems consistent with depression, post-traumatic stress disorder (PTSD), anxiety disorders, somatoform disorders, severe social stress, and acculturation difficulties in every generation.  Elderly and adolescent males were described as the most affected and in need of immediate services. | - | Psychology |
| 14 | WOS | **Psychometric Properties and Factor Structure of the Center for Epidemiologic Studies Depression Scale in a National Sample of Arab Adolescents** | Dardas, LA; Xu, HZ; Shawashreh, A; Franklin, MS; Wainwright, K; Pan, W | Issues Ment Health Nurs | 2019 | Jordan  USA | Cross-sectional study | Testing the psychometric properties and validity of the CES-D depression scale in Jordanian Arab adolescents | 13–17 y | Jordan | 3,292 adolescents representing all regions of the country — urban, suburban and rural | Construction of depression in these adolescents differed from that of the CES-D; a 2-factor structure is more appropriate.  Adolescents' had difficulty in distinguishing between depressive affect symptoms, somatic symptoms, and interpersonal problems. | CES-D | Nursing  Psychology |
| 15 | PubMed | **Cross-cultural examination of measurement invariance of the Beck Depression Inventory-II** | Dere J, Watters CA, Yu SC, Bagby RM, Ryder AG, Harkness KL. | Psychol Assess. | 2015 | Canada | Cross-sectional study with self-questionnaire | Establishing the cross-cultural validity of the Beck Depression Inventory-Second Edition assessment tool | M age = 18.14 (Chinese-Can.)  M age = 18.21 (European-Can.) | Chinese origin (native or 2nd gen.)  and European-Can. | Canadian (Ontario) undergraduate students of Chinese (n=933) and European-American (n=933) origin | The BDI-II had strong measurement invariance across both culture and gender.  Students of Chinese origin scored higher than students of European origin on cognitive symptoms of depression. | BDI-II | Psychology |
| 16 | Psycinfo | **The cross-ethnic variations in the prevalence of headache and other somatic complaints among adolescents in Northern Israel** | Genizi, Jacob; Srugo, Isaac; Kerem, Nogah C. | The Journal of Headache and Pain | 2013 | Israel | Cross-sectional comparative study using self-administered questionnaires | To examine the inter-ethnic variation in the prevalence of headache in a nonclinical sample of adolescents in northern Israel and its association with other somatic disorders. | 15–16 y | Jewish and Arab Israelis | 2,088 10th graders attending 19 high schools in northern Israel | Prevalence of headaches:  Arabs=18.6% Jews 27.9.  F > M (regardless of ethnicity).  Associated complaints were more frequent in young people with headaches: abdominal pain, palpitations, sleep problems, and fatigue. | - | Neurology  Psychophysiology |
| 17 | Psycinfo | **Comparative analysis of integrative self-knowledge, mindfulness, and private self-consciousness in predicting responses to stress in Iran** | Ghorbani, Nima; Cunningham, Christopher J. L.; Watson, P. J.; | International Journal of Psychology | 2010 | Iran  USA | Comparative study  with assessment by 1st questionnaire completed before stress of a 20-day final examination period, followed by a checklist of symptoms and vitality measures proposed at 4-day intervals during final examinations. | To analyze different aspects of self-awareness (private self-awareness, mindfulness, and integrative self-awareness) in an attempt to explain its effect on stress resistance | M=21.63 y | Iranian | 186 Iranian university students | Only mindfulness was a negative predictor of symptoms. | 20 symptom checklist | Psychology |
| 18 | PubMed | [**Phenomenology and beliefs of patients with Dhat syndrome: A nationwide multicentric study**](https://pubmed.ncbi.nlm.nih.gov/26142412/) | Grover S, Avasthi A, Gupta S, Dan A, Neogi R, Behere PB, Lakdawala B, Tripathi A, Chakraborty K, Sinha V, Bhatia MS, Patjoshi A, Rao TS, Rozatkar A. | Int J Soc Psychiatry. | 2016 | India | Cross-sectional multicenter study | To assess the phenomenology and associated beliefs in patients with Dhat syndrome. | M age at symptom onset = 23.9 y; M age = 28 y | India | 780 male patients above 16 years of age in 15 centers | Dhat syndrome is a distinct clinical entity seen throughout India, with its own characteristics | Dhat Syndrome Questionnaire | Psychiatry |
| 19 | PubMed | [**Comorbidity in patients with Dhat syndrome: a nationwide multicentric study**](https://pubmed.ncbi.nlm.nih.gov/25904237/) | Grover S, Avasthi A, Gupta S, Dan A, Neogi R, Behere PB, Lakdawala B, Tripathi A, Chakraborty K, Sinha V, Bhatia MS, Pattojoshi A, Rao TS, Rozatkar A. | J Sex Med. | 2015 | India | Cross-sectional multicenter study | To assess comorbidities in patients with Dhat syndrome in several centers across India. | Age at onset: 23.9 (8.5)  M = 28.1 | India | 780 patients above 16 yr in centers | Comorbidities:  -depression (20.5%)  neurotic, stress-related, and somatoform disorders (20.5%)  -50% have comorbid sexual dysfunction | ICD-10 | Gynecology  Reproductive Medicine  Urology |
| 20 | WOS | **Context and Timing Matter: Language Brokering, Stress, and Physical Health** | Guan, ASS; Weisskirch, RS; Lazarevic, V | J Immigr Minor Health | 2020 | USA | Cross-sectional study with self-questionnaire | Exploring the dynamic relation between language brokering (LB) and well-being among students from different backgrounds | M=23.13 y | USA,  Origin:  Latino 33.5%.  Europ-Am 32  Asia 19.6% Other/multiple  14.9% | 559 students from universities in California | LB perception as a role reversal in families or as a source of stress was associated with higher levels of somatic symptoms.  F > M | SSS-8 | Public Health  Social Sciences |
| 21 | PubMed | **Depression and somatic symptoms in Japanese and American college students: Negative mood regulation expectancies as a personality correlate** | Hamamura T, Mearns J. | Int J Psychol | 2019 | Japan  USA | Cross-sectional comparative study | To compare the association between depression and somatic symptoms in a non-Western country (Japan) and a Western country (USA), and to examine the relation of these 2 variables to expectations about negative affect regulation (NMRE): beliefs about a person's ability to improve negative moods | 18–30 y M=19.4 (US) M=19.77 (J) | Japanese and American | 155 Japanese students and 176 American students. | Depression was significantly correlated with somatic symptoms in both countries, with no effect of gender or culture.  Somatic symptoms higher in Americans.NMRE and somatic symptoms were significantly correlated. NMRE explained variance in depression in both countries, but NMRE significantly explained variance in somatic symptoms only in F in both countries. | HSCL-30 | Psychology |
| 22 | WOS | **The Burden of Care: A National Survey on the Prevalence, Demographic Characteristics, and Health Problems Among Young Adult Carers Attending Higher Education in Norway** | Haugland, BSM; Hysing, M; Sivertsen, B | Front Psychol | 2020 | Norway | Descriptive cross-sectional study based on data from the SHoT2018 study | To examine the prevalence, characteristics, and health outcomes of young people who provide informal care to family members or others with physical or mental illness, addiction, or disability. | 18–25 y  M = 22 | Norway  "Immigrant" variable studied | 40,205 students in higher education | Caregiver status was associated with the following factors: being a woman, being single, having divorced parents, being an immigrant, and having financial difficulties.  % of young carers of immigrant origin: 2.5% (vs 1.2%). | SSS-8 | Cognitive Science  Psychology |
| 23 | PubMed | **The relationship between existential well-being and mood-related psychiatric burden in Indian young adults with attachment deficits: a cross-cultural validation study** | Hiebler-Ragger M, Kamble SV, Aberer E, Unterrainer HF | BMC Psychol. | 2020 | Austria, India | Comparative study | To study the influence of spirituality on the relation between insecure attachment and mood-related psychiatric burden in Indian young adults. | 18–30 y M=22 | India | 443 young Indians with a Hindu education | Anxious attachment was associated with anxiety, depression, and somatization.  Existential well-being was associated with depression and anxiety but not somatization. | BSI-18 | Psychology |
| 24 | WOS | **Perceived Ethnic Stigma Across the Transition to College** | Huynh, VW; Fuligni, AJ | Journal of youth and adolescence. | 2012 | USA | 4-year longitudinal study | To examine the evolution over time of young people's perceptions (in transit to university) of discrimination and feelings of devaluation of their ethnic group | M=17.79 | Latino origin  Europe  Asia  Other minorities | 563 young people in transition to college, grades 9-12, in 3 Los Angeles schools | -Perceived discrimination decreased over time and was associated with depressive and somatic symptoms.  -Somatic symptoms decreased over time except in Latinos=stable.  -Perceived devaluation increased over time and led to symptoms only in ethnic minorities  - F > M in grade 12  - In 12th grade, young people from Asian backgrounds reported significantly higher levels of somatic symptoms than other ethnic minorities. | CES-D | Behavioral Sciences  Pediatrics  Psychology |
| 25 | PubMed | **Long-term mental health in unaccompanied refugee minors: pre- and post-flight predictors** | Jensen TK, Skar AS, Andersson ES, Birkeland MS. | Eur Child Adolesc Psychiatry | 2019 | Norway | 5-year longitudinal study | To study the health trajectories of unaccompanied refugee minors (URM) to increase our knowledge of the possible pathways to well-being and integration for URM in the host country | 6 months (n = 95; M age = 13.8, 80% boys),  2 y (n = 78; M age = 16.5,)  and 5 y (n= 47; M age 20 y) | Youngs who fled to Norway | URM | M somatization at 5 years = 13.95% (7.64).  Significant association between high levels of daily hassle and somatization (not with social support or trauma after arrival). | CSSI-8 | Pediatrics Psychiatry |
| 26 | PubMed | [**Acculturation-based and everyday parent-adolescent conflict among Chinese American adolescents: longitudinal trajectories and implications for mental health**](https://pubmed.ncbi.nlm.nih.gov/23088797/) | Juang LP, Syed M, Cookston JT. | J Fam Psychol. | 2012 | USA | Longitudinal study in 3 stages separated by one year | To explore the relation between the 2 types of conflict over time and their associations with adolescent adjustment (i.e., Anxiety/somatization, loneliness, depressive symptoms, and self-esteem) | M=14.8 y | Chinese-Americans | 316 Chinese-American teenagers in 2 San Francisco schools | Everyday and acculturation conflicts were linked and evolved in parallel over time.  Family conflicts and acculturation conflicts predicted anxiety and somatization. | BSI (7 items) | Psychology |
| 27 | Psycinfo | **Discrimination and adjustment among Chinese American adolescents: Family conflict and family cohesion as vulnerability and protective factors** | Juang, Linda P.; Alvarez, Alvin A | American Journal of Public Health | 2010 | USA | Cross-sectional study using self-administered questionnaires completed independently by adolescents and their parents | To examine Chinese-American adolescents' experiences of racial/ethnic discrimination to determine how discrimination is linked to poor coping skills/adjustments (i.e., loneliness, anxiety, and somatization) and how the family context may mitigate or exacerbate these links. | Teenagers  M = 14.8 | Chinese-Americans | 181 Chinese-American teenagers and their parents in San Francisco | Greater discrimination was linked to greater somatization.  Family conflict and family cohesion did not affect somatization. | BSI | Public Health |
| 28 | PubMed | [**Patient Health Questionnaire: Greek language validation and subscale factor structure**](https://pubmed.ncbi.nlm.nih.gov/22901833/) | Karekla M, Pilipenko N, Feldman J. | Compr Psychiatry. | 2012 | Cyprus | Cross-sectional study | To assess the reliability, validity, and factor structure of the Greek translation of the Patient Health Questionnaire (PHQ) | M=23 y | Cyprus | 520 Cypriot and Greek-speaking university students. | PHQ allows for the assessment of somatization/somatoform symptoms.  The PHQ demonstrated good reliability, validity, and appropriate factor structure. | PHQ  Psychiatric Diagnostic Screening Questionnaire | Psychiatry |
| 29 | WOS | **Somatization in Children and Adolescents: practical implications** | Karkhanis, DG; Winsler, A | Journal of Indian Association for Child and Adolescent Mental Health : JIACAM. | 2016 | USA | Literature review | To review the literature on somatic complaints in childhood and adolescence, describe somatic symptoms, biological and environmental influences, and discuss gender differences, cultural factors, and possible cognitive interventions. | - | International | Children and teenagers | Common: headaches, stomach aches, joint pain, then muscle pain, vomiting, gastrointestinal disorders.  Stress is a contributing factor.  F > M  Several studies reported a somatic expression of psychological problems in Asian societies. In Asian societies, the body is holistically integrated with emotions: the cultural norm is to show emotion and expressions of distress through the body. | - | Child Psychiatry |
| 30 | PubMed | **Cultural variation in temporal associations among somatic complaints, anxiety, and depressive symptoms in adolescence** | Kim JHJ, Tsai W, Kodish T, Trung LT, Lau AS, Weiss B. | J Psychosom Res. | 2019 | USA  Vietnam | Comparative longitudinal study | To examine the longitudinal relations between these 3 domains of internalizing symptoms in 3 different ethnocultural samples of adolescents. | M=15.6 – 15.8 y | Americans, Vietnamese-Americans, and Vietnamese | 10th and 11th grade students  304 European-Americans, 420 Vietnamese-Americans and 717 Vietnamese | There was a cultural difference in the temporal sequence of onset of internalized symptoms:  -For Vietnamese-American and Vietnamese adolescents, the most consistent relationship was that somatic complaints predicted increased anxiety.  -For European American adolescents, anxiety symptoms consistently predicted an increase in depressive symptoms  -Anxiety and depressive symptoms predicted each other bidirectionally in Vietnamese and Vietnamese American adolescents. | YSR | Psychiatry |
| 31 | PubMed | [**Psychological features of North Korean female refugees on the MMPI-2: latent profile analysis**](https://pubmed.ncbi.nlm.nih.gov/23730825/) | Kim SH, Kim HK, Lee N. | Psychol Assess. | 2013 | USA  Norway  Nether-lands  Sweden | Cross-sectional study with latent class analysis in a first psychological examination | To examine profile heterogeneity with the Minnesota Multiphasic Personality Inventory-2nd Edition (MMPI-2; Butcher, Dahlstrom, Graham, Tellegen, & Kaemmer, 1989) in the North Korean refugee women population | Age 18–69 y  M = 34.6  but analysis with age covariate | Korea (North) | North Korean refugee women (N = 2163) living in a center in Hanawon | The analysis shows that a higher age increased the risk of being in class 3 or 4, compared to class1.  Age = risk factor.  *Class 1 (non-clinical), Class 2 (demoralized), Class 3 (somatized) and Class 4 (detached). | MMPI-2 | Psychology |
| 32 | WOS | **The Reliability and Validity of the Korean Version of the Mood and Feelings Questionnaire for Depression in Youth: A Cross-Cultural Perspective** | Kim, H; Kim, K; Kim, JW | Child Psychiatry Hum Dev | 2020 | Korea | Cross-sectional study | To assess cross-cultural differences in depressive symptoms and the validity of the Korean version of the Mood and Feelings Questionnaire (MFQ) | 7–19 y  M = 14.2 y | Korea | 464 children and adolescents in the child psychiatry department of Seoul Hospital | No cultural differences were seeb in the clinical manifestations of depression in young people from Western countries and Korea. | MFQ-C | Pediatrics  Psychiatry |
| 33 | PubMed | **PTSD of rape after IS ("Islamic State") captivity** | Kizilhan JI. | Arch Womens Ment Health | 2018 | Germany, Iraq | Retrospective cross-sectional study | To describe the prevalence and nature of PTSD symptoms in Yezidi women reporting rape during IS captivity  To describe the comorbidity of other psychological disorders  To examine associated risk factors | 18–38 y (M = 23.72) | Yezidis (Syria, Iraq) | 296 Yezidi women rape survivors living in Germany since January 2016 as part of a project to support women and children held hostage by the IS | 67% had somatoform disorders, 53% depression, 39% anxiety, and 28% dissociation  PTSD: from 39%–57%. | Semi-structured interview of the DSM | Psychology  Women's Health |
| 34 | WOS | **Adolescents' Stigma Attitudes Toward Internalizing and Externalizing Disorders: Cultural Influences and Implications for Distress Manifestations** | Lau, AS; Guo, SS; Tsai, W; Nguyen, DJ; Nguyen, HT; Ngo, V; Weiss, B | Clin Psychol Sci | 2016 | USA | Cross-sectional comparative study | To examine predictors of stigma attitudes and emotional behavior problems common among Vietnamese-American and European-American youth. | M=15,6 y | Vietnamese-Americans  European-Americans | 1,224 adolescents in grades 10 and 11, in 3 cohorts formed over 3 academic years (2011-2013), in 10 high schools | Vietnamese-American youth were less tolerant of internalizing representations of psychopathology and more tolerant of externalizing psychopathology than European-American adolescents.  Greater tolerance of internalizing disorders in families with interdependent values compared to externalizing disorders  Stigma towards internalizing disorders was associated with lower self-reported internalizing symptoms, and stigma towards externalizing symptoms with lower externalizing symptoms. | YSR | Psychology |
| 35 | PubMed | **Morality in clinical space: treatment of youngsters with functional somatic symptoms in a Western clinical context** | Laursen SS, Dehlholm-Lambertsen B, Stenager E, Johannessen H. | Anthropol Med. | 2019 | Denmark | Longitudinal (ethnographic) study and investigation of 2 specific cases | Explore the moral implications of treating young people with functional somatic symptoms. To discover the cultural and moral values that clinical practice directs and upholds, and the implications this has for the assessment and management of illness. | 9–16 y  No M  2 case reports of 14-year-old | Denmark | 10 boys and 11 girls aged 9 to 16, hospitalized in pain clinic  Followed from Feb 2013 to Sept 2014 | Decentered management of physical pain, focusing on self-awareness and body awareness, on lifestyle | - | Anthropology  Medicine |
| 36 | WOS | **The role of childhood generalized anxiety in the internalizing cluster** | Lee, KS; Vaillancourt, T | Psychological Medicine | 2020 | Canada | Retrospective longitudinal study over a 7-year interval, based on a cohort (McMaster Teen Study) studied since 2008 with responses from young people and parents | To examine the intra-individual temporal structuring of the development of internalizing symptoms and the impact of risk factors (gender, ethnicity, socio-economic indicators, bullying victimization, child abuse) on these symptoms | 11–17 y | Canada | 703 students in Grade 5 from 51 schools | Somatization reported from the age of 13.  Anxiety and depression were predictors of somatization.  Risk factor: abuse  F > M  According to parental reports, bidirectional effects between depression and somatization.  Ethnicity negatively predicted the intersection of anxiety and somatization.  White and high-income parents report lower levels of internalized symptoms in their children. | BASC-2 (7 items) | Psychology |
| 37 | WOS | **The Psychological Problems of North Korean Adolescent Refugees Living in South Korea** | Lee, YM; Shin, OJ; Lim, MH | Psychiatry Investig | 2012 | South Korea | Cross-sectional comparative study | To assess the mental health of North Korean adolescent refugees residing in South Korea | 13–22 y (R)  M=16.3 (R)  11-17 y (C)  M=13,07 (Controls) | Korea (North) | 102 North Korean refugees at Hangyeore Middle and High School in South Korea  and 766 middle school students in Anseong | Higher levels of somatic symptoms (56.09 vs 52.9), social withdrawal, depression/anxiety, thinking, attention, ... decreased sociability, school performance, ... | Child Behavior Checklist | Psychiatry |
| 38 | Psycinfo | [**The prevalence of somatoform disorders in internal medicine outpatient departments of 23 general hospitals in Shenyang, China**](https://web-b-ebscohost-com.ezproxy.u-paris.fr/ehost/viewarticle/render?data=dGJyMPPp44rp2%2fdV0%2bnjisfk5Ie46bdIr66vTLCk63nn5Kx95uXxjL6nrkevrq1KrqiuOLWwsFG4p7c4v8OkjPDX7Ivf2fKB7eTnfLuvr0i0rLJRpOLfhuWz5Iqk2uBV7un3gKTq33%2b7t8w%2b3%2bS7Sq6nsEWurrFOtaOuSK%2bc5Ifw49%2bMu9zzhOrq45Dy&vid=65&sid=a36e9ca0-f6fe-4fb9-b404-daec343beeab@pdc-v-sessmgr06) | Liu, Li; Bi, Bo; Qin, Xiaoxia; Wei, Shengnan; Wang, Wei; Li, Yueling; Jin, Qiu; Ai, Li; Phillips, Michael R.; Dong, Guanghui | General Hospital Psychiatry | 2012 | China | Cross-sectional study with a 2-step assessment (interview with the extended Chinese version of the general health questionnaire and a structured clinical interview for DSM-IV-TR axis I disorders) | To assess the prevalence, demographics, and risk factors of somatoform disorders in the internal medicine departments of 23 randomly selected general hospitals in Shenyang, China. | M=45 y but subgroup 15–24 analyzed | China | 5,312 participants from outpatient departments of internal medicine | No somatoform disorders found in the 15-24 age group. | GHQ (12 items) | Hospitals  Psychiatry |
| 39 | WOS | **The Role of Psychological Inflexibility and Mindfulness in Somatization, Depression, and Anxiety Among Asian Americans in the United States** | Masuda, A; Mandavia, A; Tully, EC | Asian American Journal of Psychology | 2013 | USA | Cross-sectional study with online self-questionnaire | Investigating the link between psychological rigidity and mindfulness in relation to somatization, depression, and anxiety | 18-31 y  M age 19.89 y | Asian Americans | 116 university students (Southeast USA) of various Asian heritages | Both regulatory processes were related to somatization, depression, and anxiety after controlling for age and gender.  Greater psychological rigidity was associated with greater somatic, depressive, and anxiety symptoms.  Greater alertness was associated with lower somatic, depressive,and anxiety problems. | BSI 18 | Psychology |
| 40 | PubMed | **The long-term impact of cancer: Evaluating psychological distress in adolescent and young adult cancer survivors in Switzerland** | Michel G, François C, Harju E, Dehler S, Roser K. | Psychooncology | 2019 | Switzerland | Cross-sectional study | To assess psychological distress in cancer survivors by questionnaire (assessing anxiety, depression, somatization, and a global severity index)  and to compare distress levels with controls and to describe sociodemographic and cancer-related characteristics associated with psychological distress. | 16–25 y | Switzerland | 160 young cancer survivors | Young people with an immigrant background had higher levels of somatization | BSI | Neoplasms  Psychology |
| 41 | WOS | **Reconnecting Homeless Adolescents and Their Families: Correlates of Participation in a Family Intervention** | Milburn, NG; Klomhaus, AM; Comulada, WS; Lopez, SA; Bath, E; Amani, B; Jackson, J; Lee, A; Rice, E; Semaan, A; Kim, BKE | Prevention Science | 2020 | USA | Longitudinal study over 1 year with 3 stages of interviews between adolescent and parent conducted by a professional, over 5 sessions | To identify factors influencing participation in family behavioral interventions in families of adolescents living away from home | 12–17 y  M= 14,7 | USA | Adolescents (and family) in Los Angeles and San Bernardino, who lived away from home (a shelter, hotel, street, or with someone other than their parents) for > 2 nights in the past 6 months, were not away from home for more than 6 months, and were at a transition point to reconnect with their family; (i.e., had a parent who was willing to reunite with the ado.) | Better adherence (follow-up) among adolescents who reported more symptoms of depression, anxiety, somatization, OCD, phobias, and psychotic symptoms.  But from the therapists' point of view (measured by WAI = satisfaction with the therapeutic alliance), they had a better alliance with white/European-American parents and families with low BSI scores including the somatization subscale in parents  = working with more distressed or minority families seemed more difficult for therapists. | BSI | Science |
| 42 | PubMed | **Somatization and Coping in Ethnic Minority Recruits** | Nakkas C, Annen H, Brand S. | Military medicine | 2019 | Switzerland | Cross-sectional descriptive study | To study somatization levels and coping styles in (military) recruits of different ethnocultural backgrounds in Switzerland. | 18–26 y | Switzerland, Turkey, Balkan, other minorities | 740 male recruits in the Swiss army | Recruits in the "Other" group composed of various ethnic minorities reported significantly higher levels of somatieation than the group of native Swiss citizens. | SCL-90 | Military Medicine |
| 43 | WOS | **Somatization disorder among adolescents in southeast Nigeria: a neglected issue** | Nwokocha, ARC; Chinawa, JM; Onukwuli, V; Ubesie, A; Ndukuba, A; Chinawa, AT; Aniwada, E; Uwaezuoke, S | Int J Ment Health Syst | 2017 | Nigeria | Cross-sectional descriptive study | To determine the pattern and types of somatization disorder among adolescents attending secondary schools in southeastern Nigeria. | 10–19 y  M=16.36 | Nigeria | 485 youths from mixed schools (stratified random sampling) from 4 secondary schools in southeastern Nigeria. | High prevalence 62.5%.  Average age of adolescent with somatization = 16 years  Head-related symptoms (51.4%); body (54%), both (43.3%)  Males reported more head-related symptoms. | ESS (enugu somatization scale) | Mental Health |
| 44 | Psycinfo | **Racial microaggressions and daily well-being among Asian Americans** | Ong, Anthony D.; Burrow, Anthony L.; Fuller-Rowell, Thomas E.; Ja, Nicole M.; Sue, Derald Wing | Journal of Counseling Psychology | 2013 | USA | Longitudinal descriptive study via a questionnaire completed daily online for 14 days | To present data on the prevalence and psychological correlates of everyday racial microaggressions that reflect the experience of Asian Americans. | 16–20 y M=18.14 | Asian Americans | 152 first-year college students in New York | 78% reported racial microaggressions during the 2-week study period. Increased microaggressions predicted increases in somatic symptoms and negative outcomes. | Physical Symptom Checklist (7 it) | Psychology |
| 45 | Psycinfo | **Impact of childhood exposure to psychological trauma on the risk of psychiatric disorders and somatic discomfort: Single vs. multiple types of psychological trauma** | Park, Subin; Hong, Jin Pyo; Bae, Jae Nam; Cho, Seong-Jin; Lee, Dong-Woo; Lee, Jun-Young; Chang, Sung Man; Jeon, Hong Jin; Hahm, Bong-Jin; Lee, Young Moon; Seong, Sujeong; Je Cho, Maeng | Psychiatry Research | 2014 | South Korea | Cross-sectional interview study | To examine whether childhood exposure to several types of potentially traumatic events (PTEs) versus a single type of PTE was associated with a higher prevalence of psychiatric disorders and greater somatic discomfort in Korean adults. | 18–74 y  with PTE before 18  with class 18–34 y | Korea | 6,027 people | Exposure to several types of PTE was strongly associated with somatoform disorder (symptoms of fatigue, pain, mobility-related)  Somatoform disorder associated with anxious personalities and chronic symptoms | EQ-5D (Korean version of the EuroQOL 5D) | Psychiatry |
| 46 | PubMed | **Medically unexplained somatic symptoms: diagnostic and treatment issues on the Indian subcontinent** | Prakash S, Mandal P. | Am J Psychiatry | 2014 | India | Case report | To discuss the diagnosis and treatment of medically unexplained symptoms around a case of Dhat syndrome | 17 y | India | 1 male, 17 years old, living in New Delhi | Consultation of different professionals: somatician, ayurvedic doctor, psychiatrist  Culturally inappropriate treatment proposal. Misunderstanding between professional and patient who does not feel recognized.  Treatment strategies: aim to correct patient's misconceptions, improve lifestyle | - | Psychiatry |
| 47 | PubMed | **Cultural identity and mental health: differing trajectories among Asian and Latino youth** | Rogers-Sirin L, Gupta T. | J Couns Psychol. | 2012 | USA | 3-year longitudinal study | To explore the trajectories of mental health symptoms (withdrawal/depression and somatic symptoms) and how ethnic identity and American identity, as two distinct processes of identity development, affect mental health symptoms and whether these relationships are moderated by ethnic group: Asian or Latino. | M=15.7 y (at recruitment) | 1st and 2nd generation Asian and Latino migrants | 163 1st and 2nd generation Asian (n = 76) and Latino (n = 97) adolescents, recruited in the 10th grade, living in the USA | Somatic and depressive symptoms decreased over time for both groups.  Ethnic identity was associated with lower levels of somatic symptoms for Asian, but not Latino youth. American identity was not associated with reduced levels of somatic symptoms or withdrawal/depression for either group. | YSR | Psychology |
| 48 | Psycinfo | **Racial/ethnic differences in the prevalence of internalizing symptoms: Do Latin-American immigrant show more symptomatology than Spanish native-born adolescents?** | Romero-Acosta, Kelly; Penelo, Eva; Noorian, Zahra; Ferreira, Estrella; Domènech-Llaberia, Edelmira | Journal of Health Psychology | 2014 | Spain | Cross-sectional comparative study using self-administered questionnaires | To compare the prevalence of internalizing symptoms between Spanish and Latin American adolescents in Spain | 13–16 y  M=13,9 | Spain  Latin American immigrants | 834 Spanish adolescents from the city of Rubi and 159 immigrant adolescents from Latin America | No significant difference: Sp 39%, LA 37.3%.  Most frequent symptom = headache  F > M and this difference was more pronounced in Spanish adolescents. + More depressive symptoms in Latin-Americans | SSQ | Psychology |
| 49 | PubMed | **Clinical characteristics of psychogenic nonepileptic seizures across the lifespan: An international retrospective study** | Sawchuk T, Asadi-Pooya AA, Myers L, Valente KD, Restrepo AD, D' Alessio L, Homayoun M, Bahrami Z, Alessi R, Paytan AA, Kochen S, Taha F, Lazar LM, Pick S, Nicholson TR, Buchhalter J. | Epilepsy Behav. | 2020 | Canada  USA  Iran  Brazil  Venezuela  England  Argentina | Retrospective multicenter study | To study semiological, demographic, and historical risk factors according to the age of onset of PNES in a large cohort from different countries. | 4–58 y  M = 27 | Iran  Brazil,  Venezuela,  Canada,  Argentina  USA | Natives | Female predominance was associated with adolescence (85/122, 70%) and adults (190/270, 70%).  More severe PNES attacks in adults than younger people. | - | Behavioral Sciences  Neurology |
| 50 | PubMed | [**Cross-cultural findings on community violence exposure and internalizing psychopathology: comparing adolescents in the United States, Russia, and Belgium**](https://pubmed.ncbi.nlm.nih.gov/23129249/) | Schwab-Stone M, Koposov R, Vermeiren R, Ruchkin V. | Child Psychiatry Hum Dev. | 2013 | USA | Cross-national comparative study | To study cross-cultural differences in the relation between community violence and psychopathology | 14–17 y | United States  Belgium  Russia | 3,309 adolescents aged 14-17 from urban communities in the US (N = 1,343), Belgium (N = 946) and Russia (N = 1,009). | Levels of psychopathology increased with the severity of exposure to violence and those who were abused reported the highest levels of depressive symptoms, anxiety, and somatization. | BASC  behavior assess-ment system for children | Pediatrics  Psychiatryy |
| 51 | WOS | **Culture beats gender? The importance of controlling for identity- and parenting-related risk factors in adolescent psychopathology** | Seiffge-Krenke, I; Persike, M; Besevegis, E; Chau, C; Karaman, NG; Lannegrand-Willems, L; Lubiewska, K; Rohail, I | J Adolesc | 2018 | Germany  Greece  Peru  Turkey  France  Poland  Pakistan | Cross-national comparative study | To analyze the effects of gender and culture on the psychopathology of adolescents in 7 different countries | M=15 y | France  Germany  Turkey  Greece  Peru  Pakistan  Poland | 2,259 adolescents | Highest internalized symptom scores: Turkey and Greece, followed by France and Poland.  Lowest: Germany, Peru, and Pakistan.  Overall M > F with higher levels of internalizing symptoms, but not in to all countries (different scores in France, Peru, Pakistan, and Poland and similar scores in Germany, Turkey, and Greece). | YSR | Pediatrics |
| 52 | WOS | **Adolescents' somatic complaints in eight countries: what influence do parental rearing styles have?** | Seiffge-Krenke, I; Sattel, H; Cavdar, D; Oncu, B | Eur Child Adolesc Psychiatry | 2020 | Germany  England  Turkey | Cross-national comparative study | To analyze the associations between parenting styles and adolescent body complaints in different cultural contexts | 14–16 y  M=15.33 | Argentina  France  Germany  Greece  Pakistan  Peru  Poland  Turkey | 2,415 adolescents from 8 countries | Levels of body complaints vary by country:  fewer: Greece, Turkey, Argentina.  more: Peru, Pakistan, Germany.  Gender difference varies by country.  F > M  Expression of distress through body complaints is associated with parental child-rearing style:  - Increased complaints: mothers' psychological control, anxious supervision of mothers  Reduction of complaints :  - paternal support  - less paternal psychological control. | YSR | Pediatrics  Psychiatry |
| 53 | PubMed | **Transcultural differences of psychologically traumatised children and adolescents** | Shrestha AK, Özlü-Erkilic Z, Popow C, Ohmann S, Akkaya-Kalayci T. | Neuropsychiatr. | 2019 | Austria | Retrospective comparative study | To analyze the cross-cultural differences of psychologically traumatized children and adolescents with and without a migration background. | 4–18 y  M= 12.2 | Austria  and Turkish immigrant background | 200 cases of patients in the child psychiatry department in Vienna with a diagnosis of trauma-related disorder | -No significant difference in prevalence of somatization (A=39% T=32%),  Slight difference in the type of post-trauma symptoms:  A: + sleep and weight loss problems  T: + behavioral problems  -Patients of immigrant origin + adherent to treatment. |  | Neurology  Psychiatry |
| 54 | PubMed | **Alone, but protected? Effects of social support on mental health of unaccompanied refugee minors** | Sierau S, Schneider E, Nesterko Y, Glaesmer H. | Eur Child Adolesc Psychiatry. | 2019 | Germany | Cross-sectional comparative study | To analyze the effects of perceived social support (by family, peers, and/or adult mentors) in URMs, with subgroup analyses between URMs with and without family contact.  To analyze the effect of social support on the relation between stressful life events and mental health. | 14–19  M= 17.3 | Syrians and Afghans living in Germany | 105 male URM refugees | Prevalence of somatic symptoms = 24.8% (SSS-8 >= 12)  Interaction between stressful life events and somatic symptoms but no predictive relation. | SSS-8 | Pediatrics  Psychiatry |
| 55 | Psycinfo | **The role of acculturative stress on mental health symptoms for immigrant adolescents: A longitudinal investigation** | Sirin, Selcuk R.; Ryce, Patrice; Gupta, Taveeshi; Rogers-Sirin, Lauren | Developmental Psychology | 2013 | USA | Longitudinal study with 3 stages at 12-month intervals | To explore trajectories of internalization of mental health symptoms (depression, anxiety, and somatic symptoms) | M = 16.20 y | 45% Latin America  23% Asia  16% Africa or Caribbean  12% Other or mixed  1% Middle East | 332 1st and 2nd generation immigrant adolescents residing in urban areas (NY), attending 10th grade | Decreased internalizing problems during high school years.  Greater exposure to acculturative stress predicted significantly more withdrawal, somatic, and anxiety/depression symptoms.  F > M (somatic sympt.) only for 2nd generation | YSR | Pediatrics  Psychology |
| 56 | PubMed | [**Loneliness and its association with psychological and somatic health problems among Czech, Russian and U.S. adolescents**](https://pubmed.ncbi.nlm.nih.gov/27146137/) | Stickley A, Koyanagi A, Koposov R, Blatný M, Hrdlička M, Schwab-Stone M, Ruchkin V. | BMC Psychiatry. | 2016 | Sweden  Japan  Spain  Norway  Czech Republic  USA | Cross-sectional comparative study of school survey data (SAHA) | To examine factors associated with adolescent loneliness in 3 historically and culturally different countries - the Czech Republic, Russia, and the United States and to determine whether adolescent loneliness is associated with worse psychological and somatic health. | 13-15 y | Russia  Czech Republic  USA | 2,205 Czech teenagers, 1995 Russians and 2,050 Americans | Loneliness was associated with higher risk of headaches, anxiety, anddepressive symptoms.  Loneliness was correlated with poorer adolescent health in all countries. | 7 items "somatic symptoms" | Psychiatry |
| 57 | Psycinfo | **Psychosomatic problems and countermeasures in Japanese children and adolescents** | Tanaka, Hidetaka; Terashima, Shigenori; Borres, Magnus P; Thulesius, Olav | BioPsychoSocial Medicine | 2012 | Japan  Sweden | Comparative study | To present an overview of a study on mental health in Japanese children compared with Swedish children. | 10–15 y  No M | Japan  Sweden | 742 Japanese and 1,120 Swedish children from public schools | Japanese children had significantly higher scores for physical symptoms and psychiatric disorders than Swedish children.  These were more frequent in adolescents than pre-adolescents in Japan.  Most frequent pb in Japan = orthostatic intolerance.  Japanese more strongly influenced by school-related than home-related stress. | - | Psychosomatic Medicine  Social Medicine |
| 58 | PubMed | **Somatic symptoms and internalizing problems in urban youth: a cross-cultural comparison of Czech and Russian adolescents** | Tingstedt O, Lindblad F, Koposov R, Blatný M, Hrdlicka M, Stickley A, Ruchkin V. | Eur J Public Health. | 2018 | Sweden, Norway, Czech Republic | Cross-sectional comparative study with  self-assessment survey | To examine cross-cultural and gender differences in the association between somatic complaints and internalizing problems among young people in the Czech Republic and Russia, as part of a social and health assessment study in the Czech Republic and Russia. (SAHA) | 12–17 y | Czech Republic Russia | Czech (N = 4,770) and Russian (N = 2,728) teenagers from different schools | Association between somatic symptoms and internalizing problems.  The level of internalizing problems increased with that of somatic complaints. | Scale with 9 items | Epidemiology  Public Health |
| 59 | WOS | **Longitudinal Relations between Emotion Restraint Values, Life Stress, and Internalizing Symptoms among Vietnamese American and European American Adolescents** | Tsai, W; Weiss, B; Kim, JHJ; Lau, AS | J Clin Child Adolesc Psychol | 2019 | USA | Longitudinal study over 6 months in 2 phases | To examine emotion restraint values and their interactions with life stress to predict internalizing symptoms across time in Vietnamese-American (VA) and European-American(EA) adolescents | M=5.5 y | Vietnamese-Americans  European-Americans | Teenagers (321 VA, 304 EA) in 10th and 11th grade at 10 different high schools in California | In EA teens: emotional restraint was associated with higher somatic symptoms  In VA teens: emotional restraint did not predict somatic symptoms, anxiety, or depression | YSR | Pediatrics  Psychology |
| 60 | WOS | **Time Period and Birth Cohort Differences in Depressive Symptoms in the US, 1982-2013** | Twenge, JM | Social indicators research | 2015 | USA | Retrospective comparative study | To study the trends in depressive symptoms in American adolescents and adults in the United States between 1982 and 2003 | one section on teenagers | USA | - | High school students in the 2010s (compared to the 1980s) reported more somatic symptoms of depression (e.g., sleep, thinking and memory problems; shortness of breath) and were twice as likely to have seen a professional for mental problems. | - | Social Sciences |
| 61 | WOS | **Providing manualized individual trauma-focused CBT to unaccompanied refugee minors with uncertain residence status: a pilot study** | Unterhitzenberger, J; Wintersohl, S; Lang, M; Konig, J; Rosner, R | Child Adolesc Psychiatry Ment Health | 2019 | Germany | Longitudinal (pilot, uncontrolled) study over 6 months | To examine trauma-focused cognitive-behavioral therapy (TF-CBT) for URMs with PTSD, involving their professional caregivers (i.e. social workers in child and adolescent welfare settings) | M= 17.1 | Afghanistan (73%) | 26 participants in a university psycho-therapeutic outpatient clinic in Germany | Good effectiveness  Improvement in physical health problems after therapy. | PHQ 15 | Adolescent Psychiatry  Child Psychiatry |
| 62 | PubMed | [**Mental Health Outcomes for Youth Living in Refugee Camps: A Review**](https://pubmed.ncbi.nlm.nih.gov/27729500/) | Vossoughi N, Jackson Y, Gusler S, Stone K. | Trauma Violence Abuse. | 2018 | USA | Systematic review including 20 studies | Systematic review of mental health outcomes of refugee/displaced youth residing in refugee camps. | 3–22 y | Countries where the camp is located :  Iraq, Croatia, Uganda, Sweden, Bosnia... | Young refugees | 7 studies on somatic symptoms.  Refugees had more than natives, and they increased after arrival in camp for 41% of young people in a camp in Sweden  Headaches, stomach pain, fainting, dizziness, weakness... | *YSR or parents report.* | Traumatology |
| 63 | Psycinfo | **The intersection of school racial composition and student race/ethnicity on adolescent depressive and somatic symptoms** | Walsemann, Katrina M.; Bell, Bethany A.; Maitra, Debeshi | Social Science & Medicine | 2011 | USA | Retrospective study based on data from a US national longitudinal study of adolescent health | To examine how the ethnic composition of the school, as measured by the percentage of non-Hispanic white students in a school, affects depressive and somatic symptoms in a representative sample of US adolescents, and whether the association differs by ethnicity. | M = 15.9 y | USA | 18,419 pupils attending 132 secondary schools and high schools in 1994/5. | The increase in the % of white students is related to the increase in depressive symptoms among black students and high levels of somatic symptoms.  After including students' perceptions of discrimination and school attachment, the interaction between black students and racial composition at school level was no longer significant for either outcome. | List of 12 items selected by authors | Health services  Social sciences |
| 64 | Psycinfo | **The role of identity and psychosomatic symptoms as mediating the relationship between discrimination and risk behaviors among first and second generation immigrant adolescents** | Walsh, Sophie D.; Kolobov, Tanya; Raiz, Yair; Boniel-Nissim, Meyran; Tesler, Riki; Harel-Fisch, Yossi | Journal of Adolescence | 2018 | Israel | Retrospective study using data from a 2013/14 Israeli study of health behaviors in school-aged children | To examine psychosomatic symptoms and host and heritage identities as mediators of the relations between discrimination and aggressive behavior and substance use. | 11–17 y | Immigrants from the former Soviet Union and Ethiopia | 1,503 1st and 2nd generation immigrant adolescents living in Israel | Psychosomatic symptoms mediated the relations between discrimination and aggressive behavior and substance use in adolescents of immigrant origin — partially mediationfor those of ex-USSR origin and total for those from Ethiopia.  Discrimination wass linked to an increase in somatic symptoms and a decrease in the sense of identity with the host country. | HBSC psycho-somatic symptom checklist (8 it) | Pediatrics |
| 65 | Psycinfo | **Dharan or Nabhi Sarakna: A cultural syndrome presenting with unexplained medical symptoms** | Wand, Anne; Kaur, Randip | Psychosomatics: Journal of Consultation and Liaison Psychiatry | 2014 | Australia | Case report | To describe a case of Dharan or Nabhi Sarakna, a cultural syndrome prevalent in the Indian subcontinent. | 24 y | Pakistani | A woman living in Australia | Multiple physical symptoms and emotional distress.  Traditional medicine led to significant symptomatic relief. | - | Psychiatry |
| 66 | WOS | **Applying network analysis to understand depression and substance use in Indian adolescents** | Wasil, AR; Venturo-Conerly, KE; Shinde, S; Patel, V; Jones, PJ | J Affect Disord | 2020 | USA  India | Descriptive cross-sectional study by network analysis | To analyze the relation between depressive symptoms and substance use in Indian adolescents | Ado  M=13,8 y | India | 13,035 Indian adolescents in grade 9, living in Bihar | Central depressive symptoms were different from those of Westerners: feelings of failure and sad mood  Group of associated somatic symptoms: sleep disturbance, loss of appetite, lack of energy.  Low association with substance use. | PHQ-9 | Psychiatry |
| 67 | Psycinfo | **The moderating effects of gender on the associations between multidimensional hostility and psychosomatic symptoms: A Chinese case** | Weng, Chia-Ying; Lin, I-Mei; Jiang, Ding-Yu | International Journal of Psychology, | 2010 | Taïwan | Cross-sectional study with self-questionnaire | To examine the relation between multidimensional hostility and psychosomatic symptoms in Chinese culture and the effects of gender on this relation. | 18–30 y  M = 19.94 | China (Taïwan) | 398 university students recruited in Taiwan. | Gender moderated these relations  F > M (psychosomatic symptoms)  multidimensional hostility predicted psychosomatic symptoms directly and indirectly (via negative health behavior which is more frequent in men) | Psycho-somatic symptom checklist | Psychology |
| 68 | WOS | **Mental health profile and its relation with parental alcohol use problems and/or the experience of abuse in a sample of Moroccan high school students: an explorative study** | Zouini, B; Sfendla, A; Ahlstrom, BH; Senhaji, M; Kerekes, N | Ann Gen Psychiatry | 2019 | Morocco  Sweden | Cross-sectional descriptive study | Studying mental health and associated risk factors among Moroccan adolescents | 15–18 y  M = 16,56 | Morocco | 375 students in grades 10, 11 and 12 in 4 schools in Tetouan | Somatization was more frequent among youth with parental alcohol use problems or those who have been physically or psychologically abused.  In young women, exposure to violence was associated with higher levels of somatization;  In young men, the association of violence AND parental alcohol use was linked to high levels of somatic symptoms.  W > M (M=1.47 and 0.99). | BSI | Psychiatry |
